# Sustainable water–electricity cogeneration from atmospheric water

**DOI:** 10.1093/nsr/nwaf115

**Published:** 2025-03-27

**Authors:** Zhifeng Hu, He Shan, Ruzhu Wang

**Affiliations:** Institute of Refrigeration and Cryogenics, MOE Engineering Research Center of Solar Power and Refrigeration, Shanghai Jiao Tong University, China; Institute of Refrigeration and Cryogenics, MOE Engineering Research Center of Solar Power and Refrigeration, Shanghai Jiao Tong University, China; Institute of Refrigeration and Cryogenics, MOE Engineering Research Center of Solar Power and Refrigeration, Shanghai Jiao Tong University, China

## Abstract

The huge potential of atmospheric water can be effectively harnessed by integrating atmospheric water harvesting and hydrovoltaic technologies, enabling sustainable water-electricity cogeneration.

Due to explosive population growth and rapid society development, the scarcity of both water and energy is becoming increasingly serious, posing dual challenges of water and energy crises that demand urgent action. Atmospheric water, naturally replenished through the water cycle, presents a vast and untapped reservoir—holding roughly 3–4 times and 1000 times the annual global consumption of water and energy, respectively [1,2]. To exploit its huge potential, atmospheric-water-harvesting technology (AWHT) and hydrovoltaic technology (HVT) have recently flourished independently and their applications have been successfully demonstrated in diverse environments. In AWHT, freshwater is harvested from various sources (including moisture, fog and rain) through different approaches such as sorption-based moisture harvesting, cooling-based dew harvesting and fog collection. In HVT, electricity is generated through interactions between water and materials during dynamic processes (including moisture sorption, water evaporation and droplet movement) via diverse mechanisms such as streaming potential, evaporation potential, drawing potential, contact electrification and electrostatic induction.

Despite individual developments of AWHT and HVT, efficient treatment and transformation of atmospheric water both require high-performance materials, optimized interfacial interactions and enhanced heat and mass transfer. These shared needs indicate the intrinsic similarities within the multidisciplinary fields of the energy–water–air nexus and lay a foundation for their coupling. Integrating AWHT and HVT facilitates the spontaneous harvesting of water and electricity, offering sustainable access to freshwater and electricity across diverse environmental contexts (Fig. 1). As the human footprint spans various dry and humid areas (Fig. 1, top panel), targeted pathways for water–electricity cogeneration and tailored integration schemes for AWHT and HVT are necessary in regions that cover a wide range of relative humidity (RH) (Fig. 1, middle panel).

**Figure 1.**
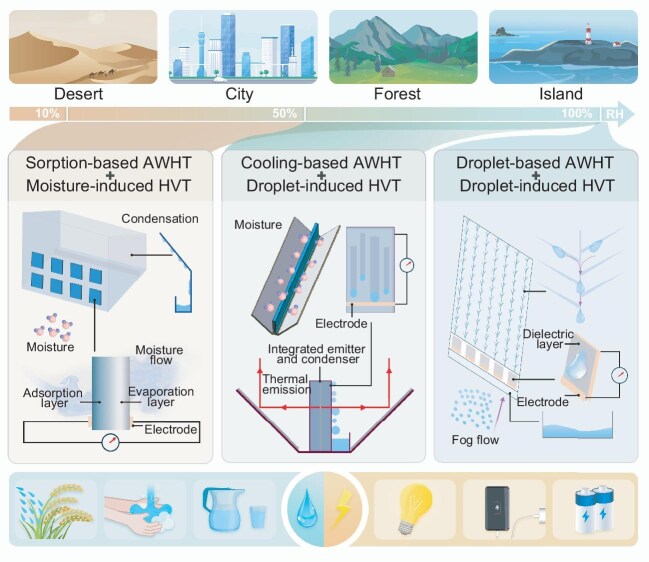
Prospect of water–electricity cogeneration from atmospheric water. Top panel: Living environments encompassing regions with various climates. Middle panel: Envisioning schemes of water–electricity cogeneration for a wide range of RH. From left to right, moisture, dew and fog are successively utilized as resources; the core components of AWHT and HVT are reproduced with permission from [4,5,9]. Bottom panel: Productions of water–electricity cogeneration.

Moisture-enabled water–electricity cogeneration is applicable to vast areas due to the ubiquitous presence of moisture, warranting significant attention for the integration of sorption-based AWHT and moisture-induced HVT [1,3]. In sorption-based AWHT, water is produced by the utilization of sorbents to sorb moisture from air and subsequently desorb it for condensation at ambient temperatures, and it is adaptable across nearly the full humidity range (∼10%–100% RH), including in extremely arid regions. Current sorption-based AWHT needs alternations between sorption and desorption in a time-based manner, leading to intermittent water production (∼1 L/(m^2^·day)) [1]. Continuous water production requires moisture to be continuously pumped into and then released from sorbents as liquid water. To meet this requirement, the sorption layer and desorption layer can be positioned on opposite sides of the material, interconnected by middle layers that feature transport channels [4]. The formed stable moisture flow enables continuous electricity generation that is caused by evaporation-induced electricity and ion diffusion [4], and the power density of the moisture-induced HVT now reaches ∼10 W/m^2^. Enhancement of water–electricity cogeneration from moisture involves the development of materials that have a higher water uptake and larger charge gradient. For scalable applications, the integration of multiple units in series or parallel connections could significantly boost the output voltage or current, and also increase water production by expanding the directional moisture-transfer capacity.

Cooling-based AWHT, in which moisture is directly condensed by cooling air to below the dew point, is inefficient in low RH but performs well in high RH (over ∼50%) environments, producing larger water volumes of up to ∼1000 L/(m^2^·day) by active refrigeration. The energy demand of refrigeration is now addressed by the adoption of passive cooling technologies such as radiative cooling, which emits heat through the transparent atmosphere window into outer space. Passive cooling-based AWHT devices with advanced radiative cooling coatings and optimized thermal management can be employed to efficiently enhance dew harvesting (∼1 L/(m^2^·day)) [5]. Droplet-induced HVT devices are easily integrated into cooling-based AWHT devices based on a condenser plate that is coated with selected polymers. The metal substrate and coatings can act as the electrode and dielectric layer, respectively, forming the bottom-electrode droplet-induced HVT devices [6]. With frequent sliding of condensed droplets on the dielectric layer, electricity is naturally generated through contact electrification and electrostatic induction [6]. Exploration of innovative structures of hydrovoltaic devices and dielectric materials with high charge-storage capability is key to enhancing electrical outputs. For instance, transistor-inspired droplet-induced HVT devices, which are constructed by placing an additional top electrode on the bottom-electrode devices [7], efficiently improve the electrical output performance (∼100 W/m^2^ per droplet). Moreover, the introduction of a lubricating layer has the effect of killing two birds with one stone, as it boosts the water-condensation rates [5] and improves the durability of the electricity-generation system [8].

A droplet-based AWHT combined with a droplet-induced HVT is promising under conditions of RH hitting ∼100%, when water droplets appear in the forms of fog and rain [9]. Tiny droplets are efficiently captured and quickly transported in droplet-based AWHT devices (yielding ∼50 L/(m^2^·h)), which are composed of well-designed meshes with optimized structures and wettability inspired by natural organisms such as cacti and desert beetles. Droplet-induced HVT devices are placed below droplet-based AWHT devices, allowing collected droplets to pass through the electricity generators before entering the water tank. When transistor-inspired droplet electricity generators are used, the energy of the impacting droplets is efficiently converted into high electrical power when the droplets make contact with the HVT devices [7]. Additionally, the use of nanomaterials such as graphene-based derivatives for fog collection meshes can generate extra electricity through the drawing potential that is created by droplets sliding on the surfaces [2].

The integration of AWHT and HVT paves a new pathway for water–electricity cogeneration from the atmosphere,

hopefully providing decentralized water resources and energy production wherever needed. This approach transcends the traditional restriction of time and space, reshaping our understanding of the energy–water–air nexus that encompasses three key elements of our human life. The produced water is safe for drinking, daily usage and even irrigation, while the generated electricity can power lights, charge smart devices or be stored (Fig. 1, bottom panel). However, future challenges still revolve around the dilute nature of atmospheric sources, as both water yield and power density remain limited at present [1,2]. Substantial enhancements in the performances of AWHT and HVT are continuously needed, which rely on material developments, device innovations and system designs. High-performance materials play a fundamental role; those with exceptional adsorption capacity and rapid kinetics are needed for higher water yields, and those with high capture and transfer efficiency of electrons or ions are required for greater electricity outputs. For devices, innovative structures are needed to fully leverage material performance, while careful regulation of heat and mass transfer is crucial to enhancing output efficiency. Importantly, drawing inspiration from nature is a vital path to push forward water–electricity cogeneration [10]. Besides, the optimized selection and combination of diverse AWHT and HVT are significant to maximize the performance across different scenarios. When moving toward systems, a modular design of the components will enhance the scalability potential, and the incorporation of efficient thermodynamic cycles and advanced energy-storage technologies is highly recommended. In short, now is the opportune moment to embrace and advance the emerging water–electricity cogeneration technology for a sustainable future.
